# Occurrence of SARS-CoV-2 in Indoor Environments With Increased Circulation and Gathering of People

**DOI:** 10.3389/fpubh.2021.787841

**Published:** 2021-11-22

**Authors:** Radka Dziedzinska, Petr Kralik, Omar Šerý

**Affiliations:** ^1^Laboratory of Neurobiology and Pathological Physiology, Institute of Animal Physiology and Genetics, Czech Academy of Sciences, Liběchov, Czechia; ^2^Elisabeth Research Institute, Brno, Czechia; ^3^Laboratory of DNA Diagnostics, Department of Biochemistry, Faculty of Science, Masaryk University, Brno, Czechia

**Keywords:** SARS-CoV-2, indoor, RT-qPCR, surface contamination, fomites, air

## Abstract

At the time of sampling (2020/2021), the number of new cases of SARS-CoV-2-positive individuals in the Czech Republic significantly exceeded the numbers in neighboring countries and in the EU. In terms of the number of deaths, the country ranked near the top of the list. Legislative orders required wearing masks indoors, disinfecting surfaces in public places, and limiting the number of people per sales area in commercial spaces. Due to an situation, most schools and shops were closed. The entire country anticipated a total lockdown. To assess the risk to public health regarding SARS-CoV-2 transmission, air and surfaces were sampled in two public places: a post office and a shopping center. Samples were also collected at the COVID-19 unit at the local hospital. Neither air nor surface samples were positive for SARS-CoV-2 virus particles in the post office or shopping center. Positive results were found in the hospital ward, with floors being the most and highest contaminated surface. Based on our results, we believe that public places do not pose a risk in relation to SARS-CoV-2 transmission, especially when epidemiological measures to reduce transmission are followed, such as wearing masks, using disinfectant or limiting the number of customers per retail establishment.

## Introduction

The SASR-CoV-2 pandemic expanded at the turn of the years 2019 and 2020, erupting from a local outbreak in Wuhan, China to spread across the entire world. The speed with which the virus traveled, combined with the ease of transmission amongst patients with mild symptoms or no symptoms at all, meant that SARS-CoV-2 infected vast numbers of people in a relatively short time. Government and hygienic services in affected countries were nearly helpless to face the virus's rapid spread within the population. A series of implemented protective measures were blindly put in place, primarily focused on restricting the population's movement. This led to the partial or complete lockdown of the commercial sector in most of the affected countries, leaving only the essential stores open. All other segments of the commercial sector remained closed without any prospect of re-opening.

However, different countries' lockdowns in the commercial sector always occurred together with other measures such as school closures, restrictions on travel between regions, cancellations of group sports, mandates for wearing masks, etc. Therefore, it is difficult to evaluate the efficacy of each individual measure. Furthermore, different countries implemented these measures to varying degrees. The main variables in the protective measures' efficacy were the motivation of citizens to adhere to the measures and adapt their behavior accordingly, as well as the impact of the virus variant itself, compounded by the overall epidemiological situation in a particular country.

The retail lockdowns were criticized as ineffective because the number of infections rose despite the retail closures. Various countries reported the same scenario. Retail representatives claimed that stores can easily restrict access and control the compliance with the hygienic measures like face mask wearing, social distancing or surface sanitization. Despite these assurances, stores remained closed, causing massive economic damage to the service and commercial sectors. Never in the past there has been such a massive restriction on the entire running of the country, involving both a restriction on the personal freedom of each individual and a restriction on the country's economy.

To help the decision-making bodies focus on effective preventive and protective measures in suppressing SARS-CoV-2 prevalence in the population, this study examined the screening of internal premises with a high movement of people to determine the risk of infection to patrons and visitors. For the purposes of the study, the central post office in Brno, Czech Republic (population 382,000) and a large shopping center in Olomouc, Czech Republic (population 100,000) were selected. The sampling was performed in the period of the SARS-CoV-2 epidemic when the percentage of positive tests exceeded 30% in the Czech Republic. The samples were taken by surface swabs from the internal environment as well as from the air. To compare the data from the publicly accessible internal premises, similar samples were collected from a local hospital's COVID-19 unit.

## Materials and Methods

### Premises Selection

The study focused on the spread of the SARS-CoV-2 virus in enclosed premises with increased crowds and unavoidable social contact. The first sampling was performed on 17 December 2020 in the evening after 6 p.m. at the central post office in Brno city with a daily turnout of 2,600 people. Sampling was performed during the holiday season when the daily percentage of newly identified cases exceeded 30% (Ministry of Health of Czech Republic; https://onemocneni-aktualne.mzcr.cz/api/v2/covid-19) ending in early January 2021. The second sampling was performed on 17 February 2021 after 6 p.m. in the Šantovka shopping center in Olomouc, in which the supermarket and food court remained open. The samples were taken predominantly from the supermarket, however other places with a higher fluctuation of people were also screened due to movements of visitors through them (Table 1). The sampling was performed when the percentage of newly identified cases remained above 30% (Ministry of Health of the Czech Republic; https://onemocneni-aktualne.mzcr.cz/api/v2/covid-19). A total of over 10,000 people visited the Šantovka shopping center on the day of sampling. Of these 59% were pedestrians and 41% arrived by car. The last sampling was performed on 6 May, 2021 in a hospital unit set up for treatment of patients who tested positive for SARS-CoV-2 (Table 2). The hospital was situated in Blansko district, ~30 km from Brno.

**Table 1:** Description of the samples collected at the post office and in the shopping center.

**Post office**		**Shopping center**	
**Type of sample**	**Number**	**Type of sample**	**Number**
Customer retrieval terminal	3	Self-service checkout display	6
Post counter	9	Shopping cart and basket handles	20^†^
Door handles	2	Escalator handrail	4
Writing desk for customers	2	Toilet door handle	5
Hands of operators	4	Hands of cashiers	6
		Elevators buttons	8
		Buttons of the parking terminal	2
Sum	20		51

† *One sample consist of swab from 3 shopping cart or basket handles*.

**Table 2:** Description of the samples collected at the COVID-19 unit.

**Location**	Type of sample^*^	**Average quantity of virus^†^**
Room 1	Patient—hands	–
	Patient—mouth	–
	Tabletop by the window	–
	Floor next to the bed	6
	Cup + mobile phone	–
	Door handles	–
	Washbasin	–
	Sink drain	–
	Bedside table	–
	Balcony handles + window handles	56
	Floor in front of Room 1	16
Room 2	Patient 1—mouth	–
	Patient 1—hands	–
	Patient 2—mouth	–
	Patient 2—hands	–
	Patient 3—mouth	4
	Patient 3—hands	–
	Tabletop	1
	Floor between the beds	–
	Edge of the bed	5
	Door handle	–
	Washbasin	3
	Sink drain	–
	Balcony handles	3
	Floor in front of Room 2	–
Examination room	PC keyboard	–
	Shared mobile phone	–
	Floor	33
Staff equipment	Shoe sole	42
	Face shield	–
	Overall	–
	Phonendoscope—ear attachments	–
Changing room	Floor	1

* *One sample was collected from each location (n = 33)*.

† *The quantity of virus (viral genomes) is related to the 5 μl of qPCR reaction*.

*–, negative*.

### Sample Collection

Two types of samples - swabs and air - were gathered in all three locations. Swab samples were taken from surfaces which come into direct contact with people's hands as well as directly from the hands themselves (Table 1). The flocked swabs (FLOQSwabs Genetics, Copan, Italy) were moistened in a 0.9% saline solution and area of ~100 cm^2^ was wiped. The swab sample was immediately placed into 600 μl of Lysis buffer (EliGene^®^ Viral DNA/RNA FAST Isolation Kit, Elisabeth Pharmacon) diluted to a 1:1 aqueous solution.

The air was sampled by the commercially available air washer LW220 (Beurer, Ulm, Germany). The principle of the air washer is based on the slow turning of a segmented wheel in water. The tank intended for the water was filled with ~4 l of the Lysis buffer (EliGene^®^ Viral DNA/RNA FAST Isolation Kit, Elisabeth Pharmacon) diluted in a 1:1 aqueous solution and the speed of the air flow was set to Maximum. The air washer was able to exchange 60 m^3^ of air per hour. This volume was determined experimentally by measuring the air flow when the machine was running at the Maximum setting.

The air washer was placed ~1 m above the floor in the location where people gathered. The main entrance door of the post office and the counter area of the supermarket were selected. On the COVID-19 unit, the air washer was placed on the window sill of a room with a covid patient. The operation time of the air washer in all three premises was 12 h, which means that 720 m^3^ flowed through the washer during each sampling. Afterwards, the remaining liquid for the air washer was collected and transferred into high-volume flasks.

### RNA Isolation

The RNA from swab samples was isolated according to the original protocol from EliGene^®^ Viral DNA/RNA FAST Isolation Kit (Elisabeth Pharmacon, Brno, Czech Republic). Two hundred microliters of the suspension with the swab was mixed with the Lysis Buffer with the Solution M added. In this step, 5 μl of internal amplification control (IAC) from the EliGene^®^ COVID-19 CONFIRM RT (Elisabeth Pharmacon) was pipetted into the suspension. The whole volume was loaded onto the column, washed, and eluted to 50 μl of Elution buffer.

The liquid from the air washer was diluted with water to the original volume (4 l). Using a vacuum manifold, four separate spin columns, and special extensions for higher volume processing, a total of 500 ml of the liquid was gradually loaded onto the columns, processing an average of 125 ml per column. IAC was applied to each column in a similar way as the RNA isolation from swabs. The washing and elution to 50 μl was performed according to the manufacturer's recommendation from the EliGene^®^ Viral DNA/RNA FAST Isolation Kit (Elisabeth Pharmacon).

### RT-qPCR Analysis

The RT-qPCR analysis was performed by the EliGene^®^ COVID-19 CONFIRM RT (Elisabeth Pharmacon) in total volume of 20 μl with 5 μl of the extracted RNA added. The qPCR protocol comprised an RT step at 55°C for 15 min, followed by the initial denaturation at 95°C for 3 min and 45 cycles of amplification at 95°C for 5 s, 55°C for 15 s, and 67°C for 15 s. The experiments were performed at CFX Touch qPCR (Bio-Rad, Hercules, CA, USA) cycler and the analysis of the results was performed by the CFX Maestro 2.0 software (version 5.0.021.0616).

To estimate an approximate number of viral particles in the samples, a simplified calibration curve was included in each run. According to the declaration of the kit's manufacturer, the positive control from the detection kit contains 10^3^ synthetic DNA molecules of the SARS-CoV-2 targets per microliter. The undiluted and 10× - and 100× -diluted positive controls were used for the construction of the calibration curve in a range 10^3^−10^1^ DNA molecules/μl, i.e., 5 × 10^3^−5 × 10^1^/qPCR reaction. The LOD of the qPCR kit was determined by the manufacturer to be 15 genomic copies per qPCR reaction. Each isolated RNA molecule was processed as a technical triplicate. Samples were considered positive if they presented as positive in at least one repeat.

## Results

### Analysis of the Central Post-office and Shopping Center Premises

A total of 71 swab samples of various surfaces, including hands of the operators/employees, were collected at the post office and in the shopping center (Table 1). All of them were found to be negative for the presence of SARS-CoV-2 virus. After 12 h of operating, the SARS-CoV-2 was detected neither in liquid from the air washer located at the post-office (*n* = 1) nor at shopping center (*n* = 1).

### Presence of SARS-CoV-2 in the Environment of the Hospital COVID-19 Unit

Altogether 33 swab samples were collected from different premises within the hospital COVID-19 unit. Eleven samples (33%) were found to be positive for the presence of SARS-CoV-2 (Table 2). The quantity of virus in the positive samples was rather low, in units or tens of viral genomes in qPCR reaction. The quantity was not absolute and served only for the rough estimation of virus particle counts. The floor and various places that come into physical contact with patients' hands were the most contaminated with the virus. Samples taken from the floor or sole were positive for the presence of virus in five cases.

The air in the room with a single patient was found to be negative for the presence of the virus.

## Discussion

Today, the potential risk of SARS-CoV-2 transmission through touching fomites is known to be negligible. In the past, a number of studies demonstrated the presence of SARS-CoV-2 virus on surfaces with a higher or lower percentage of positive samples (1–3). As simple RT-qPCR was used in most of them, they could not provide any information about the viability of viral particles or their ability to cause infection. Based on prediction models, the estimated risk of probability of SARS-CoV-2 infection from contaminated surfaces through hands was determined to be low and ranged from <10^−5^ (2) to 10^−6^ (4). In fact, the real prevalence would be probably even lower due to voluntary/ordered quarantine of an unknown fraction of positive people and due to people not coughing directly to their hands, factors which the predictive models rely upon to work (4).

In our case, positive samples were not found in the central post office nor in the public area of the mall. Negative results were obtained despite an increase of over 30% of newly identified cases per day in both sampling periods (mid-December 2020 and mid-February 2021) in the Czech Republic. At the time of sampling, the Czech Republic had implemented a requirement for wearing masks in indoor places and the mandatory disinfecting of public places. Both of these factors could have contributed to an inability to detect the virus. Only a few studies focused on monitoring the virus in different facilities others than hospitals. SARS-CoV-2 RNA appeared in 6% of samples in Italian tourist and recreational facilities (3), 8.3% of indoor and outdoor high-touch surfaces in the city of Somerville, Massachusetts (2) and in 12.3% of samples collected from public sites and households with inhabitants who had active, recovered, and negative disease statuses as confirmed by PCR tests (5). Ardura et al. (6) found low positivity only at two out of 20 urban fomites—wood playground slide and sanitizer dispenser, while any positive sample was found out of 368 samples collected from high-touch surfaces from facilities with large amounts of pedestrian traffic (7). Cycle threshold (Ct) values of qPCR positive samples ranged between 34 and 40 (3, 5, 6), which could indicate either a partially degraded RNA or a low viral load. Disinfection using recommended chemicals—particularly ethanol, chlorine-based products or hydrogen peroxide (8)—significantly reduce viral presence on surfaces, which led to the negative qPCR results obtained in previously published papers (9, 10). Recently, concerns have been raised about the futility of disinfecting surfaces in public, non-hospital places (11). In our study, the sampling of public places was always done after 6 p.m. and cleaning and disinfection of surfaces was carried out after the end of sampling. Although disinfection was carried out several times a day in common days, its omission during the experiment should have reduced the degradation of the virus and its RNA and thus increased the probability of virus capture. Despite omitting cleaning and disinfectant use, no positive sample was found. It could therefore be assumed, that the effect of disinfection on spreading the virus could be excluded in our study.

Sampling in the hospital COVID-19 unit revealed positive samples in 40% of surfaces (10 positive from 25). All patients in the rooms were in advanced stage of recovery from SARS-CoV-2 and were qPCR negative, except for one patient (Table 2). Swabs from the end stage of infections are not infrequently negative. Nevertheless, it should be noted that the patients' hands and mouths were swabbed, instead of performing a nasopharyngeal swab, which means that the results could potentially manifest as qPCR-negative results. An air sampling, performed in Room 1, revealed completely negative results. In the study by Ma et al. (12), measurement of exhaled breath condensate in SARS-CoV-2 patients showed a breath emission rate between 10^5^ and 10^7^ virus particles per hour. However, sampling of air in the rooms of these patients showed significantly lower concentrations of SARS-CoV-2 (10^3^ virus particles per hour) and positivity was proven in only one air sample of 26 (3.8%) (12). Positive air sample status varies through different studies from zero (9, 13) to more than 50% positivity (14, 15). Even if viral RNA is shown to be in the air, it says nothing about viability of SARS-CoV-2. The measuring of viable viral particles is usually not carried out or is unsuccessful (16). Nevertheless, demonstration of virions' ability to grow in a plaque assay has been proven. Lednicky et al. (17) found between 6 and 74 TCID50 units of SARS-CoV-2 viral particles per liter of air sampling in COVID-19 rooms in a hospital. Negative results of air sampled in hospitals can be explained by low virus emission, its inactivation by disinfectants, and its dilution or removal by fresh air flow as a common practice in hospital wards (12). Even then, the negative results of the air sample in our case was most probably caused by the patient's favorable condition and qPCR negativity. Any or all of the above-mentioned factors could further support this negative status.

Likewise in air, data on positivity of surface samples taken in hospital rooms with SARS-CoV-2 patients varies from higher (15, 16, 18) to very low or zero incidence (10, 19, 20). After disinfection procedures, which are subject to stricter requirements in hospitals as opposed to non-hospital facilities, the positivity of environmental samples is significantly reduced (18) or completely eliminated (9, 10, 21). In our case, SARS-CoV-2 was detected in all rooms examined, including staff rooms. Except in the room of Patient No. 2, samples from the floor (including shoe soles) were always positive despite regular cleaning and disinfection of rooms. Redmond et al. (18) also described higher contamination in floor samples compared to other high-touch surfaces. The floor is a relatively common SARS-CoV-2 contaminated surfaces in a hospital, primarily in patients' rooms (9, 16, 22). Similar to our case, viral RNA was also found in rooms other than the patients' such as the diagnostic room or the staff room (9, 12). Floor contamination may be related to subsequent contamination of shoes, which could then serve as transmission vectors of the virus (22). The reason for viral positivity on the floor is not fully clear. It could be explained by gravity and air flow causing virus particles on condensate to settle on the ground. Movement of medical staff around the ward can contribute to spreading the virus throughout the floor, including rooms without patients (22). Infrequent cleaning and the use of detergents instead of disinfectants could also play a certain role (18). The potential impact of contaminated cleaning equipment, such as the mop or floor rag, must also be taken into consideration.

The average viral load found in hospital surface swabs ranged from 1 to 56 particles per qPCR reaction (corresponding to 30–1,680 particles per swab). Similar results were obtained by Ma et al. (12) and Guo et al. (22), who estimated surface-borne viral load in hospitals to range from 10^1^ to 10^3^ viruses/cm^2^ and from 10^3^ to 10^4^ viral particles per swab. The comparison with other similar studies is however complicated due to expressing the results in Ct values (15, 16, 18) instead of counting to average viral particles. Survival of SARS-CoV2 virus on various surfaces for hours up to days was proven by *in vitro* studies (1, 23). However, high viral titres were used in the initial inoculum (10^4^-10^7^) in those studies, which does not usually reflect the real conditions. Despite evidence of SARS-CoV-2 on the surface, their growth capacity is low (17) and their ability to induce a cytopathic effect is null (24).

We are aware of the limitations of the study, which mainly lie in the one-time sampling. However, the aim of the survey was not to investigate the viability of the virus, but the real risk of potential infection of customers occurring in high concentrations in a given public place. Potential positivity in the case of sampling other public places, such as public transport, could not be refuted. However, both post-office and shopping center belong to very crowded places and moreover they enabled us to examine air comparing to, e.g., public transport.

In addition, the use of disinfectant, which was normally used several times a day, was omitted on the day of sampling in order to reduce the likelihood of degradation of the virus and its RNA and thus increase the likelihood of a positive detection. Nevertheless, no positive sample was detected at the post office or shopping center. Although negative data from both public places were submitted to the authorities, a complete lockdown of the entire country was declared 2 days after second sampling in the shopping center at the end of February. Based on our data, and previously published papers regarding low survival of the virus in the environment, its limited ability to grow in cultures, and low risk of virus transmission from fomites, we believe that closing the stores was not necessary and did not have considerable effect on minimizing the spread of the virus. In addition, compliance with anti-epidemiological regulations such as wearing masks, hand sanitization, controlling the limited number of customers per store, etc., would have been relatively easy to implement and readily observed, especially in the small local stores that were most affected by the lockdown.

## Conclusions

Despite the high incidence of newly-identified SARS-CoV-2 positive cases during the sampling in all three locations, none of the surface samples collected neither at the central post office nor in the shopping mall were positive. These findings could be probably attributed to the obligatory wearing of masks indoors, mandatory disinfection of indoor surfaces, and limited numbers of customers per sales area in the Czech Republic at the time of sampling. As expected, SARS-CoV-2 RNA was found in swabs from a local hospital. Except for patients' rooms, positive swabs were found in all rooms tested, including the staff area, despite regular cleaning and disinfection. It may be speculated that movement of staff through the unit, ineffective cleaning or disinfection procedures, or contaminated cleaning tools are potential vectors of virus transmission throughout the hospital ward.

## Data Availability Statement

The raw data supporting the conclusions of this article will be made available by the authors, without undue reservation.

## Author Contributions

RD: conceptualization, investigation, methodology, resources, writing—original draft preparation, and writing—review and editing. PK: conceptualization, funding acquisition, investigation, methodology, resources, visualization, writing—original draft preparation, and writing—review and editing. OŠ: conceptualization, funding acquisition, methodology, resources, supervision, and writing—review and editing. All authors contributed to the article and approved the submitted version.

## Funding

This work was conducted under the support of the project of Security Research of the Ministry of the Interior of the Czech Republic with the number VI04000071.

## Conflict of Interest

The authors declare that the research was conducted in the absence of any commercial or financial relationships that could be construed as a potential conflict of interest.

## Publisher's Note

All claims expressed in this article are solely those of the authors and do not necessarily represent those of their affiliated organizations, or those of the publisher, the editors and the reviewers. Any product that may be evaluated in this article, or claim that may be made by its manufacturer, is not guaranteed or endorsed by the publisher.

## References

[1] Ronca SE Sturdivant RX Barr KL Harris D. SARS-CoV-2 viability on 16 common indoor surface finish materials. HERD. (2021) 14:49–64. 10.1177/1937586721991535 33618545

[2] Harvey AP Fuhrmeister ER Cantrell ME Pitol AK Swarthout JM Powers JE. Longitudinal monitoring of SARS-CoV-2 RNA on high-touch surfaces in a community setting. Environ Sci Technol Lett. (2021) 8:168–75. 10.1021/acs.estlett.0c00875 34192125 PMC7927285

[3] Montagna MT De Giglio O Calia C Pousis C Apollonio F Campanale C. First detection of severe acute respiratory syndrome coronavirus 2 on the surfaces of tourist-recreational facilities in Italy. Int J Environ Res Public Health. (2021) 18:3252. 10.3390/ijerph18063252 33801099 PMC8004146

[4] Pitol AK Julian TR. Community transmission of SARS-CoV-2 by surfaces: risks and risk reduction strategies. Environ Sci Technol Lett. (2021) 8:263–9. 10.1021/acs.estlett.0c00966 37566313

[5] Fernández-de-Mera IG Rodríguez Del-Río FJ de la Fuente J Pérez-Sancho M Hervás D Moreno I. Detection of environmental SARS-CoV-2 RNA in a high prevalence setting in Spain. Transbound Emerg Dis. (2021) 68:1487–92. 10.1111/tbed.13817 32894654

[6] Ardura A Dopico E Fernandez S Garcia-Vazquez E. Citizen volunteers detect SARS-CoV-2 RNA from outdoor urban fomites. Sci Total Environ. (2021) 787:147719. 10.1016/j.scitotenv.2021.147719

[7] Parker CW Singh N Tighe S Blachowicz A Wood JM Seuylemezian A. End-to-end protocol for the detection of SARS-CoV-2 from built environments. Msystems. (2020) 5:e00771–20. 10.1128/mSystems.00771-20 33024053 PMC7542562

[8] Cleaning and Disinfection of Environmental Surfaces in the Context of COVID-19: Interim Guidance. World Health Organization (2020).

[9] Ong SWX Tan YK Chia PY Lee TH Ng OT Wong MSY. Air, surface environmental, and personal protective equipment contamination by severe acute respiratory syndrome coronavirus 2 (SARS-CoV-2) from a symptomatic patient. JAMA. (2020) 323:1610–2. 10.1001/jama.2020.3227 32129805 PMC7057172

[10] Wong JCC Hapuarachchi HC Arivalan S Tien WP Koo C Mailepessov D. Environmental contamination of SARS-CoV-2 in a non-healthcare setting. Int J Environ Res Public Health. (2020) 18. 10.1101/2020.05.31.20107862 33375308 PMC7797951

[11] Lewis D. COVID-19 rarely spreads through surfaces. So why are we still deep cleaning? Nature. (2021) 590:26–8. 10.1038/d41586-021-00251-4 33514939

[12] Ma J Qi X Chen H Li X Zhang Z Wang H. Coronavirus disease 2019 patients in earlier stages exhaled millions of severe acute respiratory syndrome coronavirus 2 per hour. Clin Infect Dis. (2021) 72:e652–e4. 10.1093/cid/ciaa1283 32857833 PMC7499537

[13] Luo L Liu D Zhang H Li Z Zhen R Zhang X. Air and surface contamination in non-health care settings among 641 environmental specimens of 39 COVID-19 cases. PLoS Negl Trop Dis. (2020) 14:e0008570. 10.1371/journal.pntd.0008570 33035211 PMC7577473

[14] Liu Y Ning Z Chen Y Guo M Gali NK Sun L. Aerodynamic analysis of SARS-CoV-2 in two Wuhan hospitals. Nature. (2020) 582:557–60. 10.1038/s41586-020-2271-3 32340022

[15] Razzini K Castrica M Menchetti L Maggi L Negroni L Orfeo NV. SARS-CoV-2 RNA detection in the air and on surfaces in the COVID-19 ward of a hospital in Milan, Italy. Sci Total Environ. (2020) 742:140540. 10.1016/j.scitotenv.2020.140540 32619843 PMC7319646

[16] Ben-Shmuel A Brosh-Nissimov T Glinert I Bar-David E Sittner A Poni R. Detection and infectivity potential of severe acute respiratory syndrome coronavirus 2 (SARS-CoV-2) environmental contamination in isolation units and quarantine facilities. Clin Microbiol Infect. (2020) 26:1658–62. 10.1016/j.cmi.2020.09.004 32919072 PMC7481174

[17] Lednicky JA Lauzardo M Fan ZH Jutla A Tilly TB Gangwar M. Viable SARS-CoV-2 in the air of a hospital room with COVID-19 patients. Int J Infect Dis. (2020) 100:476–82. 10.1016/j.ijid.2020.09.025 32949774 PMC7493737

[18] Redmond SN Dousa KM Jones LD Li DF Cadnum JL Navas ME. Severe acute respiratory syndrome coronavirus 2 (SARS-CoV-2) nucleic acid contamination of surfaces on a coronavirus disease 2019 (COVID-19) ward and intensive care unit. Infect Control Hosp Epidemiol. (2021) 42:215–7. 10.1017/ice.2020.416 32782056 PMC7511842

[19] Colaneri M Seminari E Piralla A Zuccaro V Filippo AD Baldanti F. Lack of SARS-CoV-2 RNA environmental contamination in a tertiary referral hospital for infectious diseases in Northern Italy. J Hosp Infect. (2020) 105:474–76. 10.1016/j.jhin.2020.03.018 32201338 PMC7156210

[20] Escudero D Boga JA Fernández J Forcelledo L Balboa S Albillos R. SARS-CoV-2 analysis on environmental surfaces collected in an intensive care unit: keeping Ernest Shackleton's spirit. Intensive Care Med Exp. (2020) 8:68. 10.1186/s40635-020-00349-5 33225382 PMC7680661

[21] Zhang S Wang C Lin M Deng Q Ye Y Li Z. Analysis of the virus contamination and disinfection effect in isolation ward of patients with COVID-19. Front Public Health. (2020) 8:486. 10.3389/fpubh.2020.00486 32984255 PMC7476240

[22] Guo ZD Wang ZY Zhang SF Li X Li L Li C. Aerosol and surface distribution of severe acute respiratory syndrome coronavirus 2 in hospital wards, Wuhan, China, (2020). Emerg Infect Dis. (2020) 26:1583–91. 10.3201/eid2607.200885 32275497 PMC7323510

[23] Kampf G Todt D Pfaender S Steinmann E. Corrigendum to “Persistence of coronaviruses on inanimate surfaces and their inactivation with biocidal agents” [J Hosp Infect 104 (2020) 246-251]. J Hosp Infect. (2020) 104:246–51. 10.1016/j.jhin.2020.01.022 32035997 PMC7132493

[24] Colaneri M Seminari E Novati S Asperges E Biscarini S Piralla A. Severe acute respiratory syndrome coronavirus 2 RNA contamination of inanimate surfaces and virus viability in a health care emergency unit. Clin Microbiol Infect. (2020) 26:1094.e1–e5. 10.1016/j.cmi.2020.05.009 32450255 PMC7243766

